# Association between glycolysis markers and prognosis of liver cancer: a systematic review and meta-analysis

**DOI:** 10.1186/s12957-023-03275-4

**Published:** 2023-12-20

**Authors:** Boqin Wang, Rong Pu

**Affiliations:** Department of Clinical Laboratory, SSL Central Hospital of Dongguan City, Dongguan, Guangdong China

**Keywords:** Hepatocellular Carcinoma, Glycolysis, Prognosis, Biomarkers, Meta-analysis

## Abstract

**Background:**

In recent years, the capacity of tumor cells to maintain high levels of glycolysis, even in the presence of oxygen, has emerged as one of the main metabolic traits and garnered considerable attention. The purpose of this meta-analysis is to investigate the prognostic value of glycolysis markers in liver cancer.

**Methods:**

PubMed, Embase, and Cochrane Library databases were searched for articles on glycolytic marker expression levels associated with the prognosis of liver cancer until April 2023. Stata SE14.0 was used to calculate the aggregate hazard ratios and 95% confidence intervals.

**Results:**

Thirty-five studies were included. The worse overall survival (OS) (*P* < 0.001), disease-free survival (DFS) (*P* = 0.001), recurrence-free survival (RFS) (*P* = 0.004), and time to recurrence (TTR) (*P* < 0.001) were significantly associated with elevated expression of glycolysis markers. Higher expression of PKM2 (*P* < 0.001), STMN1 (*P* = 0.002), MCT4 (*P* < 0.001), GLUT1 (*P* = 0.025), HK-2 (*P* < 0.001), and CA9 (*P* < 0.001) were significantly related to shorter OS. Increased levels of PKM2 (*P* < 0.001), CA9 (*P* = 0.005), and MCT4 (*P* < 0.001) were associated with worse DFS. Elevated PKM2 expression (*P* = 0.002) was also associated with poorer RFS in hepatocellular carcinoma patients. GLUT2 expression was not correlated with the prognosis of liver cancer (*P* = 0.134).

**Conclusions:**

Elevated expression of glycolysis markers was associated with worse OS, DFS, RFS, and TTR in patients with liver cancer. Therefore, these glycolysis markers could serve as potential prognostic markers and therapeutic targets in liver cancer.

**Trial registration:**

PROSPERO registration: CRD42023469645.

**Supplementary Information:**

The online version contains supplementary material available at 10.1186/s12957-023-03275-4.

## Introduction

Primary liver cancer, being one of the most prevalent malignancies worldwide, presents a substantial threat to human health [1]. According to statistical data, in the year 2012, there were approximately 780,000 newly diagnosed cases of liver cancer and 740,000 deaths attributed to this disease worldwide [2]. Hepatocellular carcinoma (HCC) is the fourth leading cause of cancer-related mortality worldwide, accounting for 80%-90% of primary liver malignancies [3, 4]. Despite the significant progress made in cancer treatment modalities, such as surgical techniques, targeted therapies, chemotherapy, and radiotherapy, the challenges of metastasis and recurrence continue to pose significant clinical challenges [5, 6]. Therefore, the identification of clinical markers that are associated with prognosis is paramount for establishing accurate diagnoses and designing individualized treatments for patients with liver cancer.

Accumulating evidence indicates that changes in cellular metabolism contribute to an increased propensity for tumor development. In cancer, a prominent hallmark involves reprogramming energy metabolism in tumor cells, which enables them to obtain the necessary energy for accelerated cellular proliferation, division, invasion, and migration [7]. One of the metabolic features of HCC cells is that glucose metabolism always terminates in pyruvate and bypasses oxidation via the Krebs cycle, which can convert pyruvate to lactate with sufficient oxygen [8]. This phenomenon, commonly referred to as "aerobic glycolysis" or the "Warburg effect," allows cells to increase glucose uptake and lactate production regardless of the presence of oxygen [9].

Numerous studies in recent years have demonstrated the effectiveness of reprogramming tumor metabolism as a new therapeutic approach to combat cancer [10]. Aerobic glycolysis is crucial for the initiation and progression of most malignancies. Therefore, inhibiting tumor cell glycolysis and interfering with the energy supply have become essential research areas in cancer treatment [11]. In vitro, in vivo, and clinical studies have reported the presence of a number of enzymes which are involved in the glycolytic pathways, including glucose transporter 1 (GLUT1), glucose transporter 2 (GLUT2), glucose transporter 4 (GLUT4), hexokinase 2 (HK- 2), monocarboxylate transporter 4 (MCT4), pyruvate kinase M2 (PKM2), stathmin 1 (STMN1), carbonic anhydrase IX (CA9), choline kinase alpha (CKA), MLX-interacting protein-like (MLXIPL), membrane-associated protein 17 (MAP17), Phosphofructokinase-2/fructose-2,6-bisphosphatase 3 (PFKFB3), phosphoglucose mutase 1 (PGM1), cyclin-dependent kinase 1 (CDK1), alanine-serine-cysteine transporter 2 (ASCT2), lactate dehydrogenase B (LDHB), homer protein homolog 1 (Homer1), tripartite motif inclusion 35 (TRIM35), phosphoglycerate kinase-1 (PGK-1), ATP-binding cassette subfamily B member 6 (ABCB6), and cyclin-dependent kinase 1 (CDC2). Among these key enzymes of glycolysis, the most widely used glycolytic markers include GLUT1, GLUT2, GLUT4, HK- 2, MCT4, PKM2, CA9, MLXIPL, PFKFB3, PGM1, CDK1, ASCT2, LDHB, PGK-1, CDC2. The glycolytic regulators STMN1, MAP17, Homer1, TRIM35, and ABCB6 are considered as potential biomarkers for the prognosis of hepatocellular carcinoma.

The GLUT family mediates glucose uptake, which transports glucose and related hexoses into the cells [12]. PKM2 is one of the major rate-limiting enzymes in glycolysis and has significant significance in the latter stages of the glycolytic pathway [13]. HK-2 catalyzes the first step of glycolysis. MLXIPL can be activated by carbohydrate metabolites, and transactivates glucose metabolism by regulating glycolysis during the circulation of sugars [14]. PFKFB3 is a metabolic enzyme that sustains glycolysis [15, 16].

Previous studies have shown that a risk signature consisting of six glycolysis-related genes can accurately predict the prognosis of HCC patients [17]. Despite the increasing number of studies on glycolysis markers, there is a need for a comprehensive review to summarize their prognostic value in liver cancer. Consequently, this meta-analysis aims to determine the relationship between glycolysis markers and the prognosis of patients with liver cancer.

## Methods

This systematic review and meta-analysis was registered on the Prospective Registry for Systematic Reviews (PROSPERO, CRD42023469645) and conducted according to Preferred Reporting Items for Systematic Reviews and Meta-analyses (PRISMA) [18, 19].

## Literature Retrieval Strategy

We searched PubMed, Embase, and Cochrane Library online databases for studies published from the establishment of each database to April 2023 that evaluated glycolysis markers concerning survival outcomes in liver cancer. The search was restricted to articles published in the English language. The search keywords comprised: “hepatocellular carcinoma”, “HCC”, “glucose transporter 1”, “GLUT1”, “monocarboxylate transporter”, “MCT4”, “hexokinase 2”, “HK2”, “pyruvate kinase M2”, “PKM2”, “Enolase 1”, “ENO1”, “L-lactate dehydrogenase B chain”, “LDHB”, “lactate dehydrogenase 5”, “LDH5”, “carbonic anhydrase 9”, “CA9”, “dihydropyrimidinase-like 4”, “DPYSL4”, “Homer protein homolog 1”, “HOMER1”, “ATP-binding cassette subfamily B member 6”, “ABCB6”, “centromeric protein A”, “CENPA”, “cyclin-dependent kinase 1”, “CDK1”, “stathmin 1”, “STMN1”, “glucose transporters”, “prognosis”, “prognostic”, and “outcome”. After removing duplicates from all identified articles, those that did not meet the inclusion criteria based on title and abstract were excluded. Then, the full text was read and evaluated carefully to identify the included literature based on the inclusion and exclusion criteria. Any differences that arose were resolved through consensus.

### Selection of Studies

The following inclusion criteria were established: (1) The diagnosis of liver cancer was based on established guidelines, such as histopathology or other relevant diagnostic criteria. (2) The study investigated the relationship between the expression levels of glycolysis markers and survival outcomes in patients with liver cancer. (3) The study was a cohort study in which patients were divided into high and low expression groups based on the expression levels of glycolytic markers. (4) The study documented the survival outcomes of HCC patients, including OS, DFS, RFS, and TTR. (5) The study presented HR and 95% CI or provided sufficient data for calculating the HR and 95% CI.

Exclusion criteria were as follows: (1) Case reports, systematic reviews, meta-analyses, letters, or conference presentations. (2) Patients included in the study with other malignancies. (3) Publications written in languages other than English. (4) Studies utilized duplicate data or analyses. (5) Studies that did not present available data. (6) The studies with a sample size of less than 20.

### Data Extraction Process

Two researchers independently extracted pertinent data from eligible studies. The extracted data for each study included the first author, publication year, population source, sample size, gender distribution, age range, glycolysis markers, detection method, duration of follow-up, and survival outcomes. OS, DFS, RFS, and TTR were the recorded survival outcomes. In instances where the original data or corresponding HR was not provided, the Engauge Digitizer v4.1 was used to extract the necessary information from the Kaplan-Meier survival curve, allowing the calculation of HR and its corresponding 95% CI [20].

### Quality Assessment of Studies

The quality of all the included studies was evaluated using the Newcastle-Ottawa Scale (NOS) [21]. The NOS scale assessed the quality of each study across three domains: selection (0–4 points), comparability (0–2 points), and exposure (0–3 points). Studies with an NOS score greater than 7 were classified as high quality, while those with a score between 5 and 7 were classified as medium quality. Studies scoring below 5 were deemed to be of poor quality [22].

### Statistical Analysis

All statistical analyses and graphical representations were performed using STATA 16.0. The association between the expression levels of glycolysis markers and OS, DFS, RFS, and TTR in patients with liver cancer was evaluated by pooled HRs and corresponding 95% CIs. The pooled HRs and corresponding 95% CIs were calculated using the random-effects model [23]. The heterogeneity among studies was assessed using the Cochran Q test and the I^2^ statistic [24]. An I^2^ value ≤ 25% indicated low heterogeneity, 25% < I^2^ < 50% showed moderate heterogeneity, and I^2^ ≥ 50% indicated high heterogeneity [24]. Subgroup analyses were conducted based on glycolysis markers and study regions. The robustness of this meta-analysis was evaluated by sequentially excluding individual studies and assessing their impact on the pooled results. Publication bias was evaluated using Begg's test and Egger's test. All statistical tests were two-tailed, and a significance level of *P* < 0.05 was considered statistically significant.

## Results

### Search Results

**Fig. 1:**
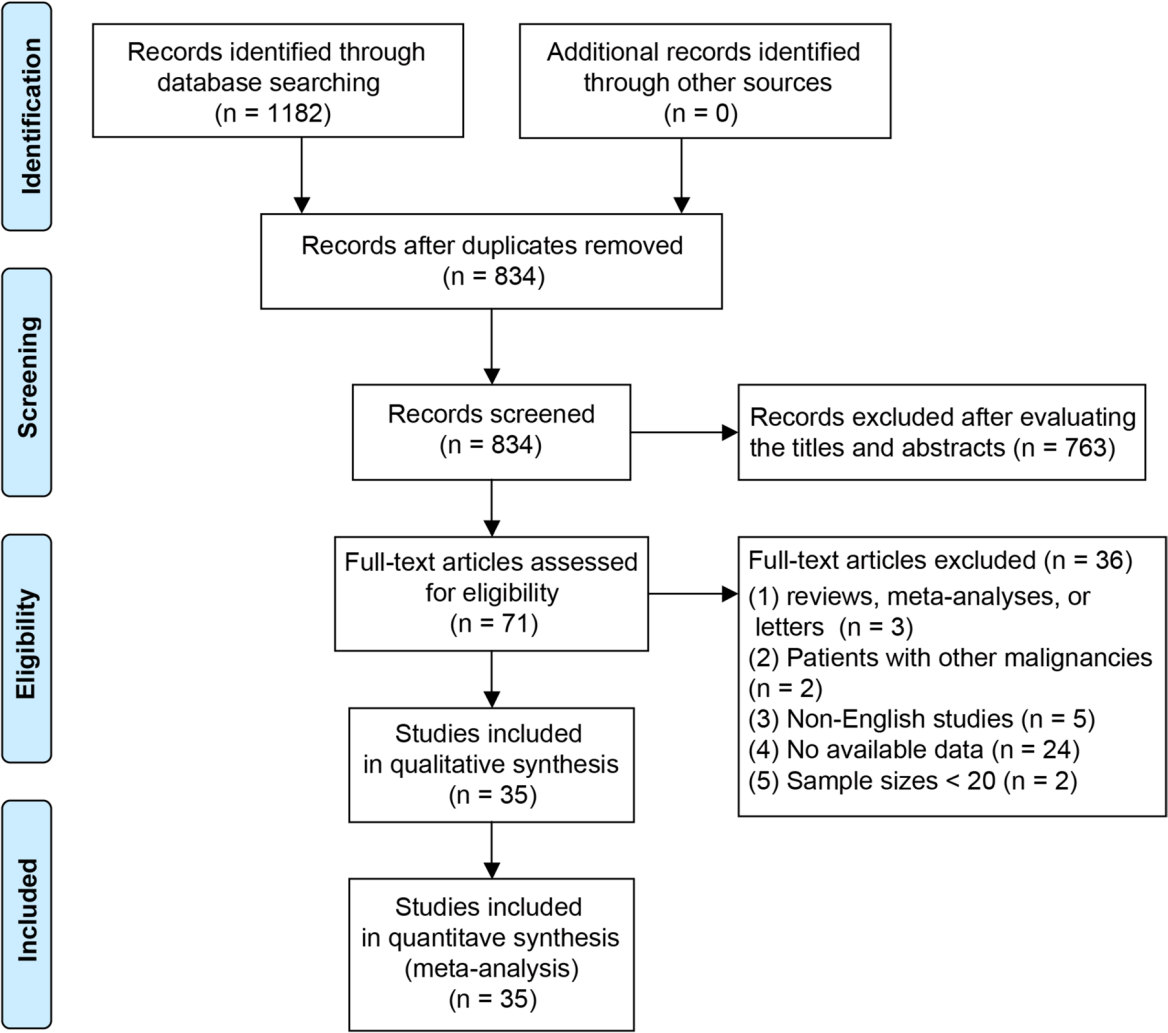
Flow diagram for selection strategy of articles in this meta-analysis

Figure 1 presents the results of the literature search and screening procedure. Initial identification yielded a total of 1182 studies. After removing duplicates, 834 investigations remained. Following a review of the titles and abstracts, 763 studies were determined to be irrelevant to the topic and were therefore excluded. The remaining 71 studies were subsequently subjected to thoroughly examining the full text. In the end, a total of 35 studies satisfied the inclusion criteria for this meta-analysis [25–59].

### Characteristics and Quality of the Included Studies

We included 35 eligible studies with a total of 5234 patients. All studies were published between 2000 and 2023, covering seven regions. A total of 22 investigations were conducted in China, 6 in Japan, 2 in Germany, 1 in South Korea, 1 in the United States, 3 in Taiwan, and 1 in Hong Kong. The sample sizes ranged from 30 to 638 individuals. In 34 investigations, tumor samples were utilized, while serum samples were utilized in only two. To measure the expression levels of glycolysis markers, 29 studies used the immunohistochemistry method, whereas 4 studies used qRT-PCR, 1 used the immunofluorescence method, and 1 used the ELISA. Glycolysis markers assessed in these studies included: MLXIPL (*n* = 1), GLUT4 (*n* = 1), PKM2 (*n* = 9), MAP17 (*n* = 1), PFKFB3 (*n* = 1), STMN1 (*n* = 3), PGM1 (*n* = 1), MCT4 (*n* = 3), GLUT1 (*n* = 2), CDK1 (*n* = 1), HK-2 (*n* = 4), CA9 (*n* = 4), Homer1 (*n* = 1), ASCT2 (*n* = 1), LDHB (*n* = 1), TRIM35 (*n* = 1), ABCB6 (*n* = 1), PGK-1 (*n* = 1), GLUT2 (*n* = 2), CDC2 (*n* = 1). The prognostic value of glycolysis markers was assessed by examining OS in 32 studies, DFS in 14 studies, RFS in 3 studies, and TTR in 3 studies. Supplementary Table 1 presented the characteristics of all eligible studies, including publication region, year, sample size, gender, age, and other relevant details.

The NOS score for the 10 studies exceeded 7, while the NOS score for the 25 studies ranged from 5 to 7.

### Correlation between Glycolysis Markers and OS in Liver Cancer

**Fig. 2:**
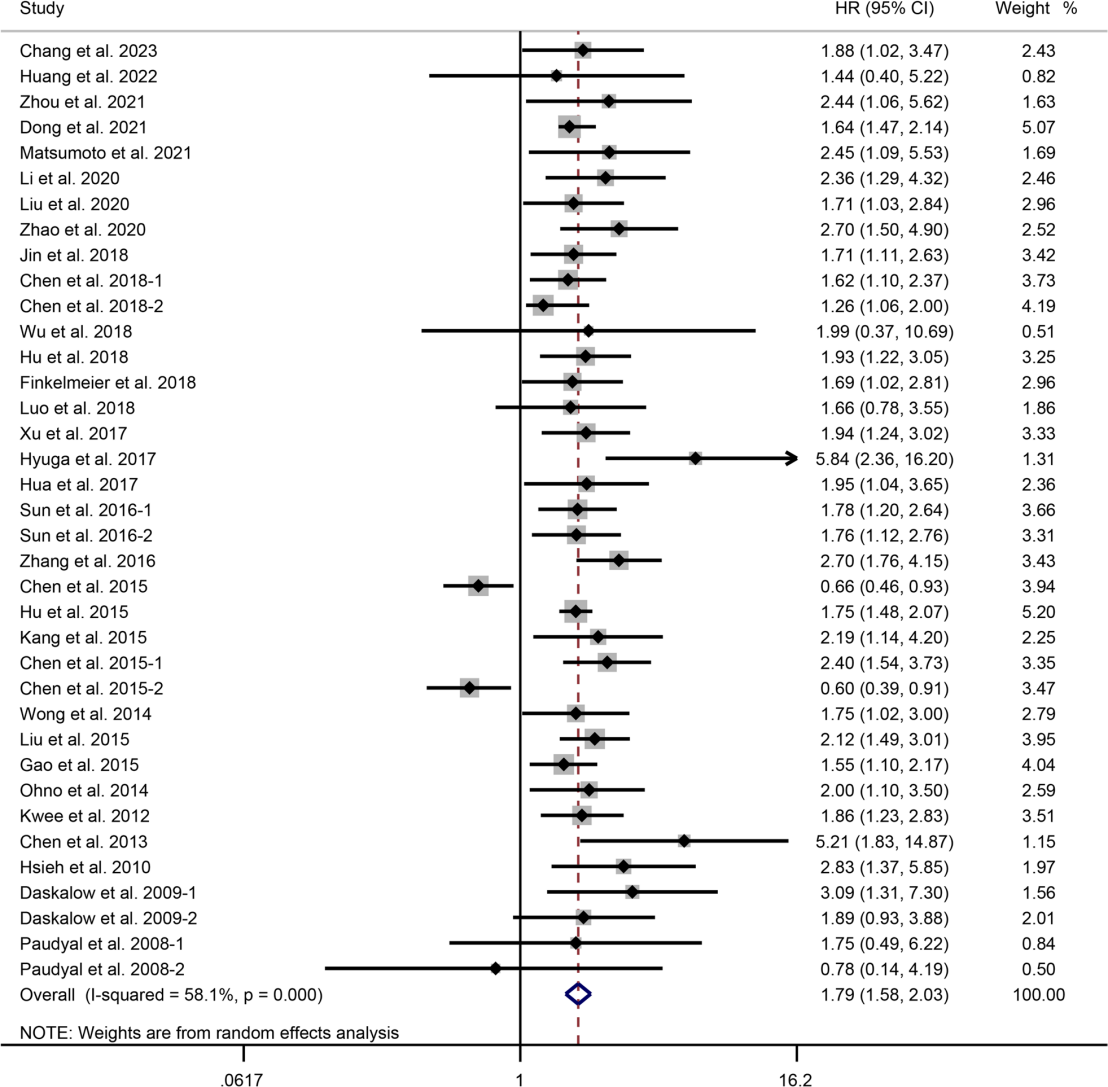
Forest plot showing the correlation between the expression levels of glycolysis markers and OS in patients with liver cancer. A random-effects model was employed. [25–39, 41–58]

In 32 studies involving 19 glycolysis markers, the association between the expression levels and OS in patients with liver cancer was investigated. The meta-analysis, using a random-effects model (I^2^ = 57.0%, *P* < 0.001), revealed that elevated expression of glycolysis markers was significantly associated with worse OS in patients with liver cancer (HR = 1.78, 95% CI: 1.58-2.01, *P* < 0.001) (Fig. 2).

Subgroup analysis based on specific glycolysis markers revealed that higher expression of PKM2 (*P* < 0.001), STMN1 (*P* = 0.002), MCT4 (*P* < 0.001), GLUT1 (*P* = 0.025), HK-2 (*P* < 0.001), and CA9 (*P* < 0.001) was significantly correlated with poor OS in liver cancer. However, elevated GLUT2 expression was not significantly associated with OS in patients with liver cancer (*P* = 0.134). The regional subgroup analysis suggested that high expression levels of glycolytic markers were associated with poorer OS in HCC patients from China (*P* < 0.001), Japan (*P* = 0.001), Germany (*P* < 0.001), and Taiwan (*P* < 0.001). Subgroup analysis of samples showed that elevated expression of glycolytic markers in tumor tissues (*P* < 0.001) and serum samples (*P* = 0.001) was associated with poor prognosis. In the assay-based subgroup analysis, high expression levels of glycolytic markers detected by IHC (*P* < 0.001) and qRT-PCR (*P* < 0.001) were associated with poorer OS in HCC patients. In addition, heterogeneity between studies could be attributable to specific glycolysis markers (Table 1). The results revealed that regions, samples, and assays were not the predominant source of heterogeneity (Table 1).

**Table 1:** Subgroup analysis of the correlation between the expression levels of glycolysis markers and OS according to the specific glycolysis markers, region, sample, and detection method

Subgroup	HR (95% CI)	Heterogeneity I^2^ (%), *P*
**Indicators:**		
MLXIPL	1.88 (1.02, 3.47)	NA
GLUT4	1.44 (0.40, 5.20)	NA
PKM2	1.93 (1.70, 2.18)	I^2^ = 0.0%, *P* = 0.704
MAP17	1.64 (1.36, 1.98)	NA
PFKFB3	2.45 (1.09, 5.52)	NA
STMN1	2.57 (1.43, 4.65)	I^2^ = 49.6%, *P* = 0.138
PGM1	1.71 (1.11, 2.63)	NA
MCT4	1.64 (1.30, 2.07)	I^2^ = 0.0%, *P* = 0.755
GLUT1	1.47 (1.05, 2.05)	I^2^ = 44.1%, *P* = 0.181
CDK1	1.99 (0.37, 10.70)	NA
HK-2	2.12 (1.66, 2.71)	I^2^ = 0.0%, *P* = 0.604
CA9	2.26 (1.47, 3.47)	I^2^ = 41.1%, *P* = 0.165
Homer1	1.66 (0.78, 3.54)	NA
ASCT2	1.76 (1.12, 2.76)	NA
LDHB	0.66 (0.46, 0.93)	NA
TRIM35	0.60 (0.39, 0.92)	NA
PGK-1	3.09 (1.31, 7.29)	NA
GLUT2	1.65 (0.86, 3.20)	I^2^ = 0.0%, *P* = 0.347
**Region:**		
China	1.66 (1.43, 1.94)	I^2^ = 67.4%, *P* < 0.001
Japan	2.37 (1.44, 3.88)	I^2^ = 28.1%, *P* = 0.234
Germany	1.95 (1.34, 2.83)	I^2^ = 0.0%, *P* = 0.493
Taiwan	2.70 (1.64, 4.45)	I^2^ = 21.5%, *P* = 0.280
South Korea	2.19 (1.14, 4.20)	NA
Hong Kong	1.75 (1.02, 3.00)	NA
US	1.86 (1.23, 2.82)	NA
**Sample:**		
Tissue	1.80 (1.57, 2.05)	I^2^ = 60.3%, *P* < 0.001
Serum	1.82 (1.29, 2.56)	I^2^ = 0.0%, *P* = 0.704
**Detection method:**		
IHC	1.80 (1.56, 2.07)	I^2^ = 63.4%, *P* < 0.001
qRT-PCR	1.80 (1.30, 2.50)	I^2^ = 0.0%, *P* = 0.885
IF	2.45 (1.09, 5.52)	NA
ELISA	1.69 (1.02, 2.81)	NA

*MLXIPL* MLX interacting protein like, *GLUT4* glucose transporter 4, *PKM2* pyruvate kinase M2, *MAP17* membrane-associated protein 17, *PFKFB3* phosphofructokinase-2/fructose-2,6-bisphosphatase 3, *STMN1* stathmin 1, *PGM1* phosphoglucomutase 1, *MCT4* monocarboxylic acid transporter 4, *GLUT1* glucose transporter 1, *CDK1* cyclin dependent kinase 1, *HK-2* hexokinase 2, *CA9* carbonic anhydrase IX, *ASCT2*, alanine-serine-cysteine transporter 2, *LDHB* lactate dehydrogenase B, *TRIM35*, tripartite motif-containing 35, *PGK-1* phosphoglycerate kinase-1, *GLUT2* glucose transporter 2, *HR* hazard ratio, *IHC* immunohistochemistry, *qRT-PCR* quantitative real time polymerase chain reaction, *IF* immunofluorescence, *ELISA* enzyme linked immunosorbent assay

### Correlation between Glycolysis Markers and DFS in Liver Cancer

**Fig. 3:**
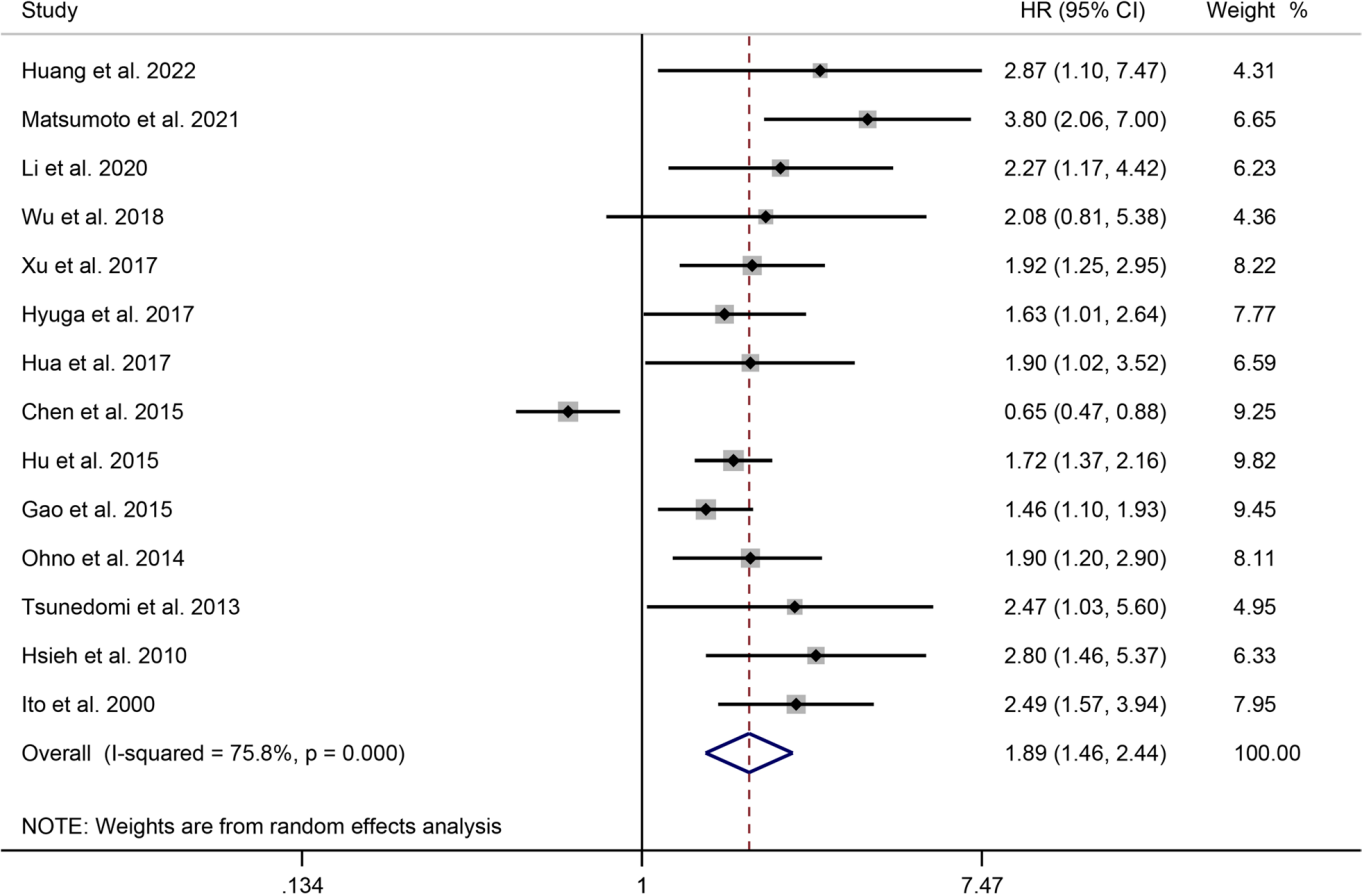
Forest plot showing the correlation between the expression levels of glycolysis markers and DFS in patients with liver cancer. A random-effects model was employed

The correlation between glycolysis markers and DFS in HCC patients was investigated in 14 studies, including 1421 patients. High heterogeneity was observed among the studies (I^2^ = 75.8%, *P* < 0.001). The pooled HR was 1.89 (95% CI: 1.17-1.81, *P* = 0.001), indicating that liver cancer patients with elevated expression of glycolysis markers had a shorter DFS (Fig. 3).

High expression of PKM2 (*P* < 0.001), CA9 (*P* = 0.005), and MCT4 (*P* < 0.001) predicted a poor DFS in patients with liver cancer, as determined by subgroup analysis based on specific glycolysis markers. The regional subgroup analysis indicated that high expression levels of glycolytic markers correlated with poorer DFS in China (*P* = 0.019), Japan (*P* < 0.001), and Taiwan (*P* < 0.001). The results of subgroup analysis based on detection methods suggested that high expression levels of glycolytic markers detected using IHC (*P* < 0.001) and qRT-PCR (*P* = 0.003) were associated with poor DFS. Furthermore, the heterogeneity between studies can be attributed to changes in glycolytic markers, rather than the study regions or detection methods (Supplementary Table 2).

### Correlation between Glycolysis Markers and RFS in Liver Cancer

**Fig. 4:**
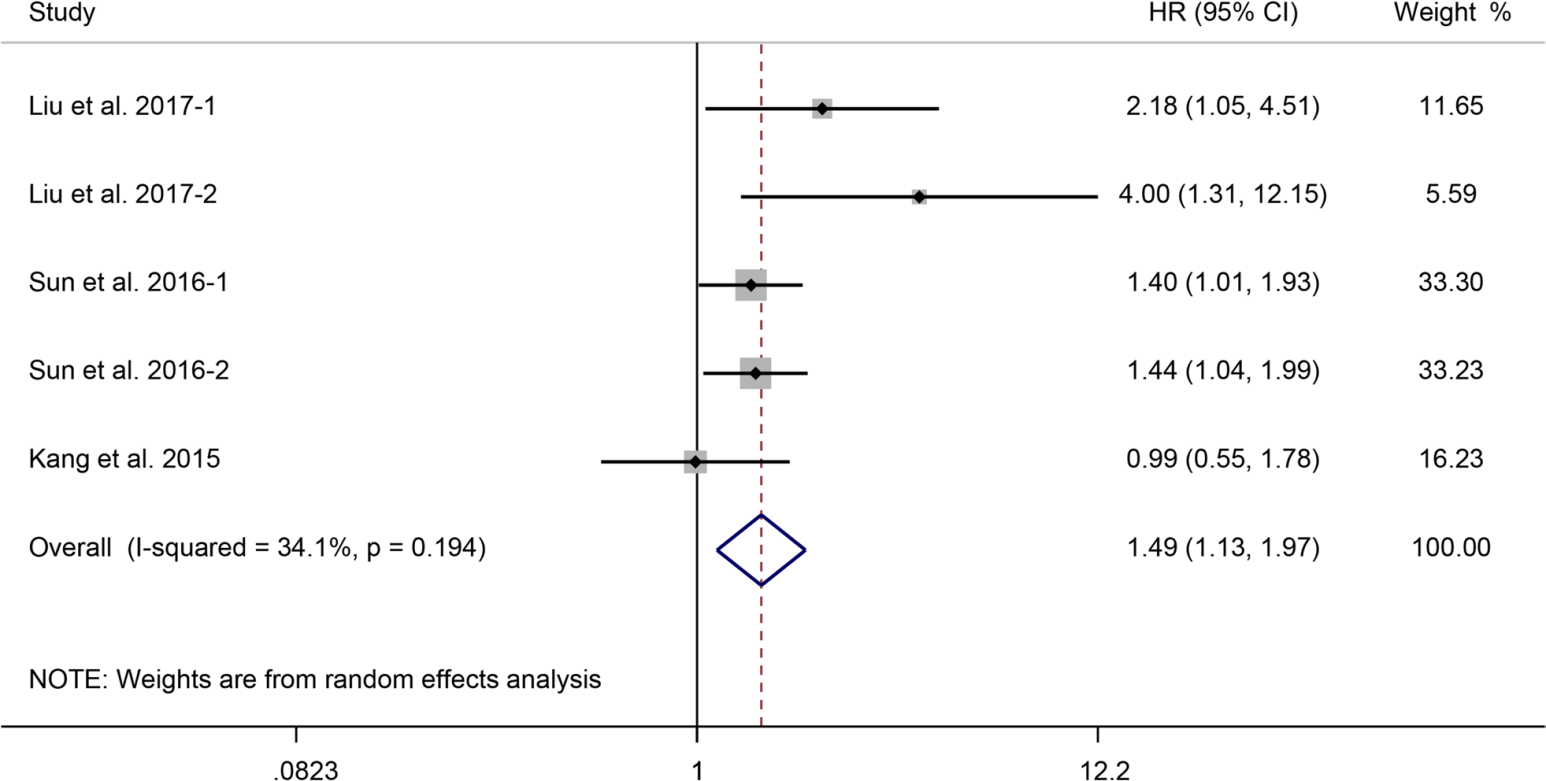
Forest plot showing the correlation between the expression levels of glycolysis markers and RFS in patients with liver cancer. A random-effects model was employed

We analyzed data from three studies, including 636 patients, to investigate the correlation between PKM2, GLUT1, ASCT2, and CA9 expression levels and RFS in patients with liver cancer. The pooled results demonstrated moderate heterogeneity (I^2^ = 34.1%, *P* = 0.194), and elevated expression levels of these four glycolysis markers were significantly associated with a shorter RFS (HR = 1.49, 95% CI: 1.13-1.97, *P* = 0.004) (Fig. 4).

A subgroup analysis based on various glycolysis markers revealed that overexpression of PKM2 (*P* = 0.002) indicated a poor RFS (Supplementary Table 3).

### Correlation between Glycolysis Markers and TTR in Liver Cancer

Using data from three studies, we examined the relationship between the expression levels of PGM1, MCT4, GLUT1, and PKM2 and TTR in HCC patients. The pooled results, with low heterogeneity (I^2^ = 0.0%, *P* = 0.814), revealed that the high expression of these four glycolysis markers in liver cancer was associated with a higher TTR rate (HR = 1.48, 95% CI: 1.25-1.75, *P* < 0.001) (Supplementary Figure 1). However, subgroup analyses could not be conducted due to the limited number of studies reporting on TTR.

### Sensitivity Analysis

The results of sensitivity analysis showed that our results were robust (Supplementary figure 2, and Supplementary figure 3).

### Publication Bias

Begg's and Egger's tests were employed to evaluate publication bias in this meta-analysis. OS (*P* = 0.059 and *P* = 0.105, respectively), DFS (*P* = 0.189 and *P* = 0.065, respectively), RFS (*P* = 0.221 and *P* = 0.307, respectively), and TTR (*P* = 0.734 and *P* = 0.070, respectively) showed no significant publication bias.

## Discussion

HCC is a global disease with significant consequences. Early detection is crucial for effective HCC management because it not only improves patient prognosis but also preserves valuable societal resources [60]. The rewiring of energy metabolism is a defining feature of cancer, with aerobic glycolysis playing a crucial role in promoting cancer cell proliferation, invasion, and migration [7, 61]. Various tumor types have been observed to exhibit dysregulation of glucose metabolism, most notably through aerobic glycolysis. This meta-analysis examined the relationship between glycolysis markers and survival outcomes in HCC patients.

Within the field of oncology, significant attention has been dedicated to studying metabolic changes in cancer. Following our inclusion criteria, we identified 36 studies examining the effect of glycolytic marker levels on the prognosis of HCC patients. Our findings demonstrated that increased expression of glycolytic markers was associated with decreased OS, DFS, RFS, and TTR in HCC patients. These results are consistent with the findings of the majority of the studies included in our analysis. In addition, subgroup analysis of distinct glycolysis markers revealed a relationship between PKM2, STMN1, MCT4, GLUT1, HK-2, CA9, and GLUT2 and the clinical outcome of HCC patients.

PKM2, a crucial rate-limiting enzyme in glycolysis, is highly active in the latter stages of the glycolytic pathway [13]. It is predominantly expressed in differentiated tissues, such as adipose tissue, lung tissue, and retinal tissue, as well as in cells with high rates of nucleic acid synthesis, such as proliferating cells, embryonic cells, and tumor cells [62, 63]. The concentration of pyruvate directly influences lactate production. PKM2 serves a vital role in tumorigenesis by catalyzing the conversion of phosphoenolpyruvate to pyruvate and releasing energy [64]. Numerous studies have reported that overexpression of PKM2 is correlated with an unfavorable prognosis and chemotherapy resistance in various tumor types [27]. Consistent with previous findings, increased PKM2 expression in HCC patients was associated with decreased OS, DFS, and RFS. In addition, PKM2-induced phosphorylation of histone H3 in HCC promotes the transcription of programmed death-ligand 1 (PD-L1) via epidermal growth factor (EGF). This leads to immunosuppression and tumor development within the HCC tumor microenvironment [65].

STMN1 is essential for regulating microtubule motility and is implicated in cancer cell division and proliferation [66]. Published studies have consistently reported that elevated STMN1 expression is associated with poorer survival in various cancers, including head and neck squamous cell carcinoma, gallbladder carcinoma, esophageal squamous cell carcinoma, breast cancer, and endometrial carcinoma. In addition, Zhang et al. demonstrated that eight glycolysis-related genes, namely AURKA (aurora kinase A), CDK1, CENPA, DEPDC1 (DEP domain containing 1), HMMR (hyaluronan-mediated motility receptor), KIF20A (kinesin family member 20A), PFKFB4 (6-Phosphofructo-2-Kinase/Fructose-2,6-Biphosphatase 4), and STMN1, are correlated with both OS and DFS in patients with HCC, which is consistent with our findings [67].

Lactic acid has been identified as a major energy source in cancer [68]. MCT4 is responsible for transporting pyruvate, lactate, and ketones as a monocarboxylate transporter [69]. It exports monocarboxylates accompanied by protons [70]. MCT4 is frequently upregulated in various malignancies, and its increased expression correlates with a poor prognosis. In liver cancer, abnormal MCT4 expression has been associated with early recurrence and a poor prognosis after radical resection [38]. This meta-analysis supports previous research findings, indicating that MCT4 can serve as both a therapeutic target and a prognostic marker for hepatocellular carcinoma [48].

The GLUT family, which consists of 14 members, plays a crucial role in the uptake of glucose and facilitates the transportation of glucose and related hexoses into cells [12]. Glucose is the primary energy source for cells and satisfies the elevated energy requirements of cancer cells involved in various biochemical processes [71, 72]. Multiple varieties of cancer have been linked to an upregulation of the glucose transporter GLUT1, which has a high affinity for glucose [73]. Amann et al. demonstrated that GLUT1 is essential for the proliferation and migration of HCC cells [74]. Consistent with previous research, our findings indicate that elevated GLUT1 expression is associated with decreased overall survival in liver cancer patients and can serve as a prognostic indicator for the disease [44]. In contrast to GLUT1, GLUT2 has a comparatively low affinity for glucose, mannose, galactose, and fructose, but a high affinity for glucosamine [75, 76]. Two investigations conducted in 2017 and 2022 demonstrated that GLUT2 is not only a negative prognostic factor in HCC but also a diagnostic imaging target for the disease [57, 77]. However, our meta-analysis revealed no correlation between the expression levels of GLUT2 and the overall survival of patients with HCC. This observation could be attributed to the limited number of studies and sample sizes in the analysis.

HK-2, the rate-limiting enzyme in the first stage of glycolysis, is essential for converting glucose to glucose-6-phosphate [78]. Overexpression of HK-2 frequently occurs in various tumors, resulting in enhanced glucose metabolism, resistance to cell apoptosis, and tumor-invading capacity. The regulatory function of HK-2 in cancer cells is complex. HK-2 overexpression initially increases glycolytic flux. HK-2 also translocates to the mitochondrial outer membrane, binds to the voltage-dependent anion channel (VDAC) porin, and inhibits apoptosis by preventing the formation of the mitochondrial permeability transition pore [79, 80]. Given HK-2's dual function in cancer cells, it is an attractive target for anticancer therapies. According to a study by Kwee et al. [55], HK-2 expression has biological and prognostic significance in HCC and may serve as an independent predictor of HCC survival. Our results also corroborate this theory, demonstrating that elevated levels of HK-2 expression are linked to reduced overall survival in HCC patients.

This meta-analysis demonstrated that elevated CA9 expression was associated with OS and DFS in HCC patients. A transmembrane protein, CA9, with an extracellular catalytic domain, CA9, is regulated by HIF-1 and plays a role in pH regulation under hypoxic conditions, such as hypoxia, acidosis, and oncogenic alterations [37]. As an adaptive response to hypoxia, the upregulation of CA9 in tumors significantly contributes to the malignant transformation of cancer and precancerous lesions [81, 82]. Hyuga et al. observed that CA9 is a crucial predictor of a poor prognosis after radical resection of liver cancer and can enhance the malignant potential of HCC cells by regulating epithelial-mesenchymal transition [41]. Genetic variation in the 3' untranslated region (3'UTR) of CA9 regulates the expression of CA9 and the progression of cancer, and serves as a novel determinant and target for HCC metastasis and prognosis, according to a study conducted in Taiwan [42].

With the development of cancer research, the metabolic reprogramming of tumors is now recognized as a promising therapeutic target, leading to significant advances in anticancer treatment. In this meta-analysis, we evaluated the effect of alterations in glycolysis markers expression on the prognosis of patients with liver cancer. The results indicated that glycolysis markers, including PKM2, STMN1, MCT4, GLUT1, HK-2, CA9, and GLUT2, can serve as potential prognostic biomarkers and therapeutic targets for liver cancer. Future research should investigate the role of glycolysis markers in the differentiation, migration, invasion, and stemness of tumor cells. These findings have significant implications for the development of novel prognostic biomarkers and the advancement of adjuvant therapies for liver cancer.

### Limitations

First, we found significant heterogeneity among studies related to OS and DFS. Despite employing random-effects models for analysis and performing subgroup analysis to investigate the sources of heterogeneity, these effects could not be completely eliminated or explained. Significant heterogeneity suggested that study results need to be treated with caution. In addition, due to the lack of standardized cutoff values for various glycolysis markers, different studies included in our analysis might have used different cutoff values, which might have a potential impact on our results. Furthermore, the majority of the patient data included in this study originated from research conducted on Asian populations. This introduces the possibility of group selection bias and limits the applicability of the conclusions to other populations. Additionally, the data selected for the meta-analysis could be subject to potential publication bias. Although neither Begg's test nor Egger's test showed significant publication bias, the majority of the studies we included reported positive results. Finally, the included studies lacked crucial information regarding survival outcomes. Even though we estimated HR and their corresponding 95% CIs by extracting data from Kaplan-Meier survival curves, it was vital to note that these calculated values were inherently less precise than those directly provided by the original studies.

## Conclusions

This meta-analysis demonstrated that the high expression of glycolysis markers was strongly associated with decreased OS, DFS, RFS, and TTR in liver cancer patients. Higher expression levels of PKM2, STMN1, MCT4, GLUT1, HK-2, and CA9 were significantly associated with reduced OS, as determined by subgroup analysis based on specific glycolysis markers. Patients with high expression levels of PKM2, CA9, and MCT4 were predicted to have a poorer DFS. Furthermore, elevated PKM2 expression was associated with reduced RFS in patients with liver cancer.

### Supplementary Information

**Additional file 1: Supplementary figure 1.** Forest plot showing the correlation between the expression levels of glycolysis markers and TTR in patients with liver cancer. A random-effects model was employed.

**Additional file 2: Supplementary figure 2.** Sensitivity analysis between the expression levels of glycolysis markers and OS.

**Additional file 3: Supplementary figure 3.** Sensitivity analysis between the expression levels of glycolysis markers and DFS (A), RFS (B) and TTR (C).

**Additional file 4: Supplementary Table 1.** Overview and characteristics of the eligible studies.

**Additional file 5: Supplementary Table 2.** Subgroup analysis of the correlation between the expression levels of glycolysis markers and DFS according to the specific glycolysis markers, region, and detection method.

**Additional file 6: Supplementary Table 3.** Subgroup analysis of the correlation between the expression levels of glycolysis markers and RFS according to the specific glycolysis markers.

## Data Availability

The datasets used and/or analysed during the current study are available from the corresponding author on reasonable request.

## Abbreviations

OS: overall survival
DFS: disease-free survival
RFS: recurrence-free survival
TTR: time to recurrence
HR: hazard ratio
CI: confidence interval
HCC: hepatocellular carcinoma
PRISMA: Preferred Reporting Items for Systematic Reviews and Meta-analyses
ENO1: Enolase 1
LDH5: lactate dehydrogenase 5
DPYSL4: dihydropyrimidinase-like 4
CENPA: centromeric protein A
MLXIPL: MLX interacting protein like
GLUT4: glucose transporter 4
PKM2: pyruvate kinase M2
MAP17: membrane-associated protein 17
PFKFB3: phosphofructokinase-2/fructose-2,6-bisphosphatase 3
STMN1: stathmin 1
PGM1: phosphoglucomutase 1
MCT4: monocarboxylic acid transporter 4
GLUT1: glucose transporter 1
CDK1: cyclin dependent kinase 1
HK-2: hexokinase 2
CA9: carbonic anhydrase IX
ASCT2: alanine-serine-cysteine transporter 2
LDHB: lactate dehydrogenase B
TRIM35: tripartite motif-containing 35
CKA: choline kinase alpha
PGK-1: phosphoglycerate kinase-1
GLUT2: glucose transporter 2
ABCB6: ATP-binding cassette subfamily B member 6
CDC2: cyclin-dependent kinase 1
Homer1: Homer protein homolog 1
NOS: Newcastle-Ottawa Scale
IHC: immunohistochemistry
qRT-PCR: quantitative real time polymerase chain reaction
IF: immunofluorescence
ELISA: enzyme linked immunosorbent assay
PD-L1: programmed death-ligand 1
EGF: epidermal growth factor
AURKA: aurora kinase A
DEPDC1: DEP domain containing 1
HMMR: hyaluronan-mediated motility receptor
KIF20A: kinesin family member 20A
PFKFB4: 6-Phosphofructo-2-Kinase/Fructose-2,6-Biphosphatase 4
VDAC: voltage-dependent anion channel
3'UTR: 3' untranslated region
