# Supplementary figures and images for: Association between glycolysis markers and prognosis of liver cancer: a systematic review and meta-analysis
Source: World J Surg Oncol. 2023 Dec 20;21:390. doi: 10.1186/s12957-023-03275-4 (PMC10731852; doi:10.1186/s12957-023-03275-4)

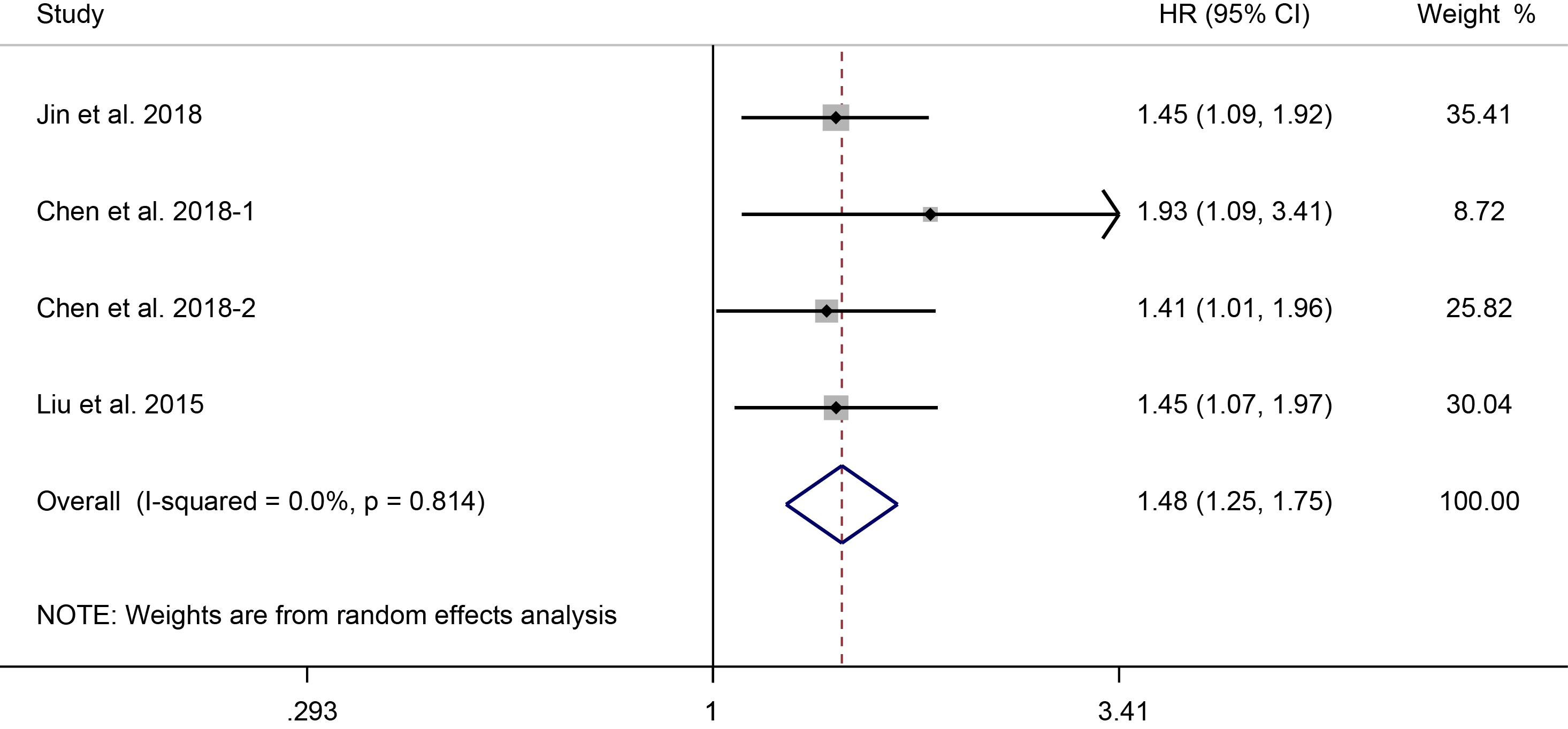

Supplement: Supplementary file 1 — Additional file 1: Supplementary figure 1. Forest plot showing the correlation between the expression levels of glycolysis markers and TTR in patients with liver cancer. A random-effects model was employed. [file 12957_2023_3275_MOESM1_ESM.png]

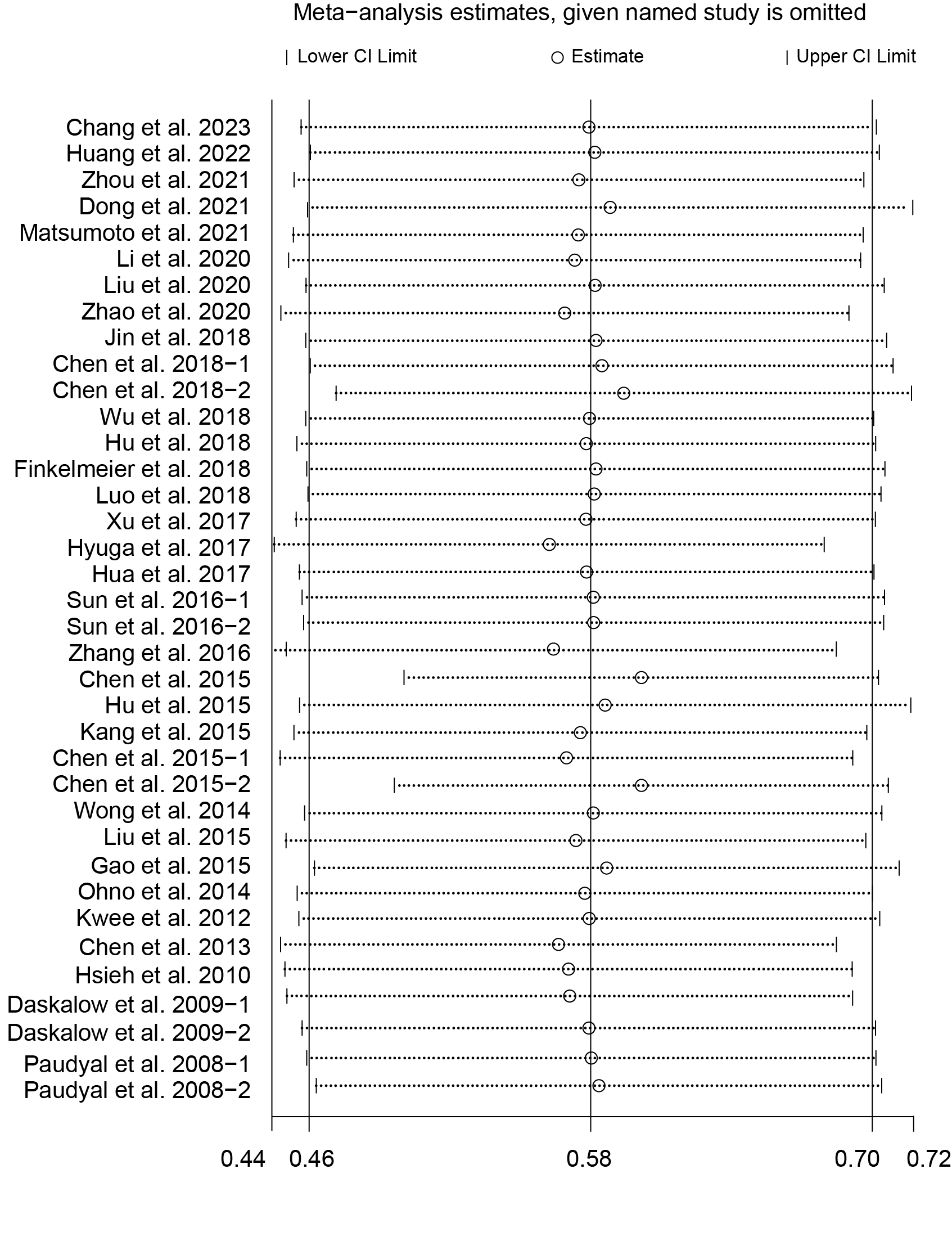

Supplement: Supplementary file 2 — Additional file 2: Supplementary figure 2. Sensitivity analysis between the expression levels of glycolysis markers and OS. [file 12957_2023_3275_MOESM2_ESM.png]

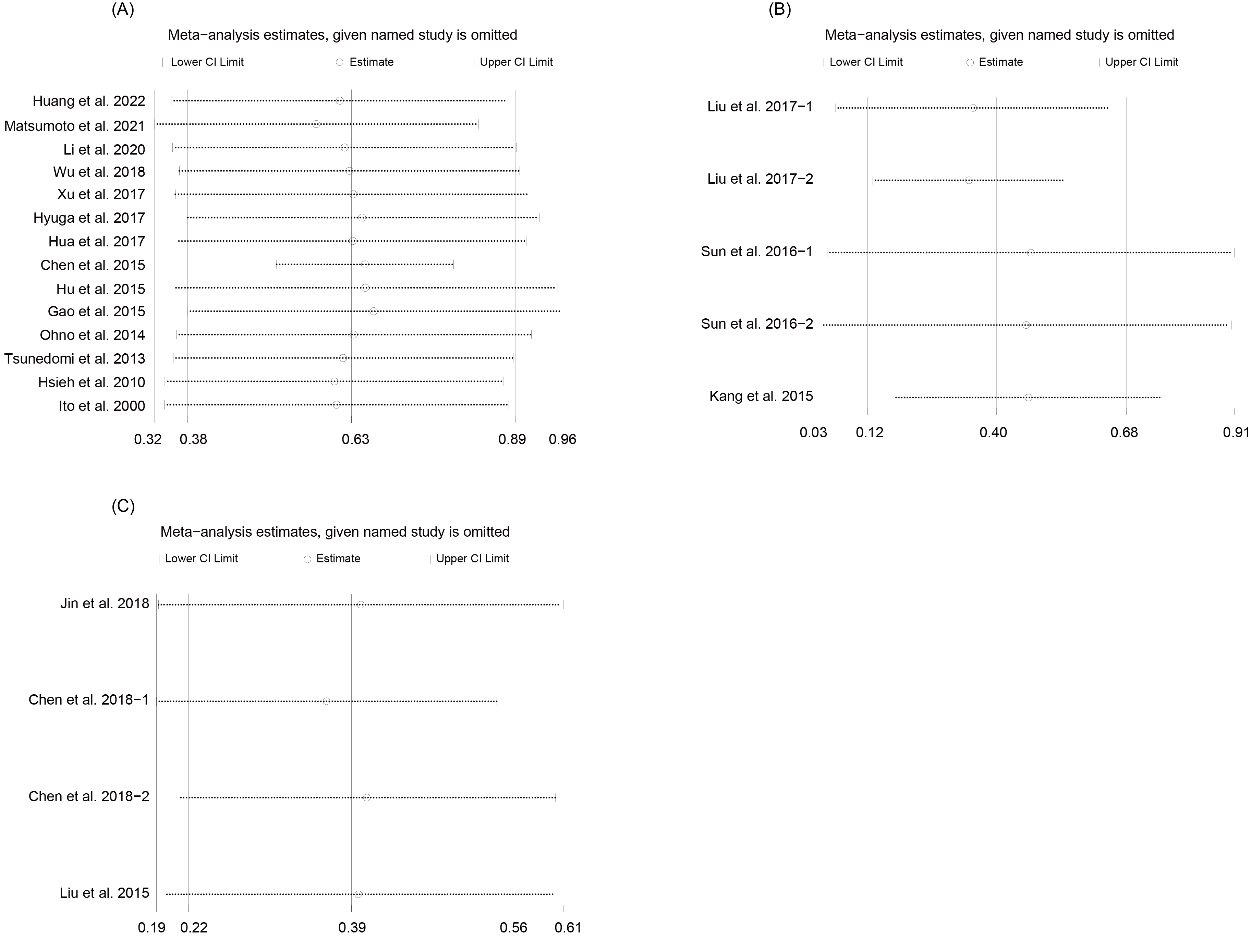

Supplement: Supplementary file 3 — Additional file 3: Supplementary figure 3. Sensitivity analysis between the expression levels of glycolysis markers and DFS (A), RFS (B) and TTR (C). [file 12957_2023_3275_MOESM3_ESM.png]
