# Supplementary material for: Association between glycolysis markers and prognosis of liver cancer: a systematic review and meta-analysis
Source: World J Surg Oncol. 2023 Dec 20;21:390. doi: 10.1186/s12957-023-03275-4 (PMC10731852; doi:10.1186/s12957-023-03275-4)
Supplement: Supplementary file 4 — Additional file 4: Supplementary Table 1. Overview and characteristics of the eligible studies. [file 12957_2023_3275_MOESM4_ESM.docx]

**Supplementary Table 1. Overview and characteristics of the eligible studies.**

| Author | Year | Region | Glycolysis marker | Patients | Gender (M/F) | Cancer | Outcome | Method | Follow-up (months) | NOS score |
| --- | --- | --- | --- | --- | --- | --- | --- | --- | --- | --- |
| Chang et al. | 2023 | China | MLXIPL | 56 | 38/18 | HCC | OS | IHC | 60 | 7 |
| Huang et al. | 2022 | China | GLUT4 | 30 | 26/4 | HCC | OS, DFS | qRT-PCR | 30 | 6 |
| Zhou et al. | 2021 | China | PKM2 | 72 | 52/20 | HCC | OS | IHC | 50 | 7 |
| Dong et al. | 2021 | China | MAP17 | 202 | NA | HCC | OS | IHC | 100 | 6 |
| Matsumoto et al. | 2021 | Japan | PFKFB3 | 99 | 73/26 | HCC | OS, DFS | IF | median 50.4 (4.3-97) | 7 |
| Li et al. | 2020 | China | PKM2 | 87 | 73/14 | HCC | OS, DFS | IHC | 100 | 7 |
| Liu et al. | 2020 | China | STMN1 | 103 | 88/15 | liver cancer | OS | qRT-PCR, wb | 60 | 5 |
| Zhao et al. | 2020 | China | PKM2 | 86 | 57/29 | HCC | OS | IHC | 36 | 8 |
| Jin et al. | 2018 | China | PGM1 | 272 | NA | HCC | OS, TTR | IHC | 100 | 5 |
| Chen et al. | 2018 | China | MCT4, GLUT1 | 213 | 182/31 | HCC | OS, TTR | IHC | mean 60.74 | 8 |
| Wu et al. | 2018 | China | CDK1 | 39 | 32/7 | HCC | OS, DFS | IHC | 60 | 7 |
| Hu et al. | 2018 | China | HK-2 | 88 | 49/39 | HCC | OS | qRT-PCR | 60 | 6 |
| Finkelmeier et al. | 2018 | Germany | CA9 | 215 | 171/44 | HCC | OS | ELISA | median 298 (1-1464) days | 7 |
| Luo et al. | 2018 | China | Homer1 | 86 | 79/7 | HBV-HCC | OS | IHC | 50 | 7 |
| Xu et al. | 2017 | China | PKM2 | 100 | 65/35 | HCC | OS, DFS | IHC | 60 | 8 |
| Liu et al. | 2017 | China | PKM2 | 219 | 160/59 | HCC | DSS, RFS | IHC | 36 | 8 |
| Hyuga et al. | 2017 | Japan | CA9 | 117 | 92/25 | HCC | OS, DFS | IHC | 60 | 9 |
| Hua et al. | 2017 | Taiwan | CA9 | 86 | NA | HCC | OS, DFS | IHC | 2500 days | 7 |
| Sun et al. | 2016 | China | GLUT1, ASCT2 | 192 | 167/25 | HCC | OS, RFS | IHC | median 56.2 (2-96) | 9 |
| Zhang et al. | 2016 | China | HK-2 | 155 | 125/30 | HCC | OS | IHC | 150 | 7 |
| Chen et al. | 2015 | China | LDHB | 75 | 67/8 | HCC | OS, DFS | IHC | median 26 (1-67) | 8 |
| Hu et al. | 2015 | China | PKM2 | 638 | 578/60 | liver cancer | OS, DFS | IHC | mean 25.9 | 7 |
| Kang et al. | 2015 | South Korea | CA9 | 225 | 169/56 | HCC | OS, RFS | IHC | median 57 (5-104) | 7 |
| Chen et al. | 2015 | China | PKM2, TRIM35 | 236 | 206/30 | HCC | OS | IHC | median 60.0 (3.0-74.0) | 6 |
| Wong et al. | 2014 | Hong Kong | PKM2 | 109 | NA | HCC | OS | IHC | 60 | 6 |
| Liu et al. | 2015 | China | PKM2 | 367 | 319/48 | HCC | OS, TTR | IHC | 60 | 7 |
| Gao et al. | 2015 | China | MCT4 | 318 | 281/37 | HCC | OS, DFS | IHC | Mean 3.41±2.50 (0.25-8.92) years | 8 |
| Ohno et al. | 2014 | Japan | MCT4 | 225 | 168/57 | HCC | OS, DFS | IHC | mean 3.6 years | 8 |
| Tsunedomi et al. | 2013 | Japan | ABCB6 | 81 | 62/19 | HCC | DFS | qRT-PCR | 72 | 5 |
| Kwee et al. | 2012 | US | HK2 | 159 | 113/46 | HCC | OS | IHC | mean 48 (0-294) | 6 |
| Chen et al. | 2013 | Taiwan | STMN1 | 34 | 23/11 | HCC | OS | IHC | median 54.2 (8.4-106.8) | 6 |
| Hsieh et al. | 2010 | Taiwan | STMN1 | 58 | 40/18 | HCC | OS, DFS | IHC | mean 41 (2-96) | 7 |
| Daskalow et al. | 2009 | Germany | PGK-1, GLUT2 | 60 | 42/18 | HCC | OS | IHC | median 28 (1-63) | 5 |
| Paudyal et al. | 2008 | Japan | GLUT2, HK-2 | 31 | 24/7 | HCC | OS | IHC | 3.37±2.26 (0.25-7.92) Years | 8 |
| Ito et al. | 2000 | Japan | CDC2 | 101 | 85/16 | HCC | DFS | IHC | mean 21.7 (5-83) | 7 |

MLXIPL, MLX interacting protein like; GLUT4, glucose transporter 4; PKM2, pyruvate kinase M2; MAP17, membrane-associated protein 17; PFKFB3, phosphofructokinase-2/fructose-2,6-bisphosphatase 3; STMN1, stathmin 1; PGM1, phosphoglucomutase 1; MCT4, monocarboxylic acid transporter 4; GLUT1, glucose transporter 1; CDK1, cyclin dependent kinase 1; HK-2, hexokinase 2; CA9, carbonic anhydrase IX; ASCT2, alanine-serine-cysteine transporter 2; LDHB, lactate dehydrogenase B; TRIM35, tripartite motif-containing 35; PGK-1, phosphoglycerate kinase-1; GLUT2, glucose transporter 2; ABCB6, ATP-binding cassette subfamily B member 6; CDC2, cyclin-dependent kinase 1; M, male; F, female; HCC, hepatocellular carcinoma; HBV-HCC, hepatitis B virus-induced HCC; OS, overall survival; DFS, disease-free survival; RFS, recurrence-free survival; TTR, time to recurrence; IHC, immunohistochemistry; IF, immunofluorescence; qRT-PCR, quantitative reverse transcription-polymerase chain reaction; wb, western blot; ELISA, enzyme-linked immunosorbent assay; NOS, Newcastle-Ottawa Scale.
