# Supplementary material for: Association between glycolysis markers and prognosis of liver cancer: a systematic review and meta-analysis
Source: World J Surg Oncol. 2023 Dec 20;21:390. doi: 10.1186/s12957-023-03275-4 (PMC10731852; doi:10.1186/s12957-023-03275-4)
Supplement: Supplementary file 5 — Additional file 5: Supplementary Table 2. Subgroup analysis of the correlation between the expression levels of glycolysis markers and DFS according to the specific glycolysis markers, region, and detection method. [file 12957_2023_3275_MOESM5_ESM.docx]

**Supplementary Table 2. Subgroup analysis of the correlation between the expression levels of glycolysis markers and DFS according to the specific glycolysis markers, region, and detection method.**

| Subgroup | HR (95% CI) | Heterogeneity I^2^ (%), *P* |
| --- | --- | --- |
| **Indicators:** | | |
| GLUT4 | 2.87 (1.10, 7.48) | NA |
| PFKFB3 | 3.80 (2.06, 7.00) | NA |
| PKM2 | 1.80 (1.48, 2.18) | I^2^ = 0.0%, *P* = 0.701 |
| CDK1 | 2.08 (0.81, 5.36) | NA |
| CA9 | 1.73 (1.18, 2.52) | I^2^ = 0.0%, *P* = 0.702 |
| LDHB | 0.65 (0.48, 0.88) | NA |
| MCT4 | 1.58 (1.24, 2.00) | I^2^ = 0.0%, *P* = 0.324 |
| ABCB6 | 2.47 (1.06, 5.76) | NA |
| STMN1 | 2.80 (1.46, 5.37) | NA |
| CDC2 | 2.49 (1.57, 3.94) | NA |
| **Region:** | | |
| China | 1.57 (1.08, 2.30) | I^2^ = 82.6%, *P* < 0.001 |
| Japan | 2.25 (1.71, 2.96) | I^2^ = 25.0%, *P* = 0.255 |
| Taiwan | 2.28 (1.46, 3.58) | I^2^ = 0.0%, *P* = 0.398 |
| **Detection method:** |  |  |
| IHC | 1.71 (1.31, 2.24) | I^2^ = 76.9%, *P* < 0.001 |
| qRT-PCR | 2.64 (1.08, 2.30) | I^2^ = 0.0%, *P* = 0.818 |
| IF | 3.80 (2.06, 7.00) | NA |

GLUT4, glucose transporter 4; PFKFB3, phosphofructokinase-2/fructose-2,6-bisphosphatase 3; PKM2, pyruvate kinase M2; CDK1, cyclin dependent kinase 1; CA9, carbonic anhydrase IX; LDHB, lactate dehydrogenase B; MCT4, monocarboxylic acid transporter 4; ABCB6, ATP-binding cassette subfamily B member 6; STMN1, stathmin 1; CDC2, cyclin-dependent kinase 1; HR, hazard ratio; IHC, immunohistochemistry; qRT-PCR, quantitative real time polymerase chain reaction; IF, immunofluorescence.
