# Supplementary material for: Association between glycolysis markers and prognosis of liver cancer: a systematic review and meta-analysis
Source: World J Surg Oncol. 2023 Dec 20;21:390. doi: 10.1186/s12957-023-03275-4 (PMC10731852; doi:10.1186/s12957-023-03275-4)
Supplement: Supplementary file 6 — Additional file 6: Supplementary Table 3. Subgroup analysis of the correlation between the expression levels of glycolysis markers and RFS according to the specific glycolysis markers. [file 12957_2023_3275_MOESM6_ESM.docx]

**Supplementary Table 3. Subgroup analysis of the correlation between the expression levels of glycolysis markers and RFS according to the specific glycolysis markers.**

| Subgroup | HR (95% CI) | Heterogeneity I^2^ (%), *P* |
| --- | --- | --- |
| **Indicators:** | | |
| PKM2 | 2.61 (1.42, 4.81) | I^2^ = 0.0%, *P* = 0.372 |
| GLUT1 | 1.40 (1.01, 1.94) | NA |
| ASCT2 | 1.44 (1.04, 1.99) | NA |
| CA9 | 0.99 (0.55, 1.78) | NA |

PKM2, pyruvate kinase M2; GLUT1, glucose transporter 1; ASCT2, alanine-serine-cysteine transporter 2; CA9, carbonic anhydrase IX; RFS, recurrence-free survival; HR, hazard ratio.
